# First record of *Spirometra* spp. eggs in fecal samples from *Panthera onca* in the Brazilian Pantanal: a One Health approach

**DOI:** 10.1590/S1984-29612025066

**Published:** 2025-11-28

**Authors:** Paul Raad, Flávio Paixão de Alencar, Richard de Campos Pacheco, Samuel Felipe Bressan, José Gabriel Gonçalves Lins, Felipe Fornazari

**Affiliations:** 1 Universidade Estadual Paulista – UNESP, Faculdade de Medicina Veterinária e Zootecnia – FMVZ, Programa de Pós-graduação em Animais Selvagens, Botucatu, SP, Brasil; 2 Instituto de Mitigação de Problemas Ambientais com Comunidades Tradicionais e Onças – IMPACTO, Poconé, MT, Brasil; 3 Universidade Federal do Mato Grosso – UFMT, Instituto de Biociências – IB, Cuiabá, MT, Brasil; 4 Universidade Federal do Mato Grosso – UFMT, Faculdade de Medicina Veterinária – FAVET, Laboratório de Parasitologia Veterinária e Doenças Parasitárias dos Animais Domésticos e Silvestres, Cuiabá, MT, Brasil; 5 Universidade Estadual Paulista – UNESP, Faculdade de Medicina Veterinária e Zootecnia – FMVZ, Departamento de Clínica Veterinária, Laboratório de Enfermidades Parasitárias dos Animais, Botucatu, SP, Brasil; 6 Universidade Estadual Paulista – UNESP, Faculdade de Medicina Veterinária e Zootecnia – FMVZ, Departamento de Produção Animal e Medicina Veterinária Preventiva, Núcleo de Pesquisa em Zoonoses – NUPEZO, Botucatu, SP, Brasil

**Keywords:** Zoonotic parasites, *Spirometra* spp., Panthera onca, coproparasitology, One Health, Parasitas zoonóticos, *Spirometra* spp., Panthera onca, coproparasitologia, Saúde Única

## Abstract

This study examines the presence of gastrointestinal parasites in fecal samples from free-ranging jaguars (*Panthera onca*) in the Pantanal biome and assesses their potential as bioindicators of environmental health at the human-animal interface using a noninvasive approach. In 2024, ten fresh fecal samples were collected from the ground at Piuval Lodge in the northern Pantanal, state of Mato Grosso, Brazil. All samples were morphologically consistent with large felids, suggesting a high likelihood of originating from jaguars, based on field evidence. Coproparasitological analysis using sedimentation and flotation techniques identified *Spirometra* spp. eggs, with a positivity rate of 100%. Additionally, 20% of the samples tested positive for *Toxocara* spp. and one sample was positive for the genus *Ancylostoma*. This study highlights that jaguars (*Panthera onca*) are a potential bioindicator of environmental health and a sentinel species in the Pantanal, thus emphasizing the interaction between wildlife, domestic animals and human activities.

## Resumo

Este estudo examina a presença de parasitos gastrointestinais em onças-pintadas (*Panthera onca*) e avalia seu potencial como bioindicadores da saúde ambiental na interface entre humanos e animais, utilizando uma abordagem não invasiva. Em 2024, dez amostras fecais frescas foram coletadas na Pousada Piuval, no Pantanal Norte, Mato Grosso, Brasil. Todas as amostras eram morfologicamente compatíveis com felinos de grande porte, sugerindo alta probabilidade de origem de onça-pintada com base em evidências de campo. A análise coproparasitológica, realizada por meio das técnicas de sedimentação e flutuação, identificou ovos de *Spirometra* spp., com taxa de positividade de 100%. Adicionalmente, 20% das amostras apresentaram positividade para *Toxocara* spp, e uma amostra foi positiva para o gênero *Ancylostoma*. Este estudo destaca a onça-pintada (*Panthera onca*) como potencial bioindicadora da saúde ambiental e espécie sentinela no Pantanal, enfatizando a interação entre a vida silvestre, os animais domésticos e as atividades humanas.

The jaguar (*Panthera onca*) is the largest felid in the Americas and occupies the top of the food chain (Quigley et al., 2017; Moraes et al., 2024). This species requires large areas with abundant prey and high-quality habitats, making it extremely sensitive to environmental disturbances. For this reason, it is considered an excellent indicator of environmental quality (Jędrzejewski et al., 2023). Currently, the species is classified as Near Threatened on the IUCN Red List, with evidence of population decline across much of its geographic range. The most robust populations are found in South America, especially in the Pantanal, one of the regions with the highest density of jaguars (Quigley et al., 2017; Kantek et al., 2021).

The One Health concept integrates human, animal and environmental health, exploring how these domains interconnect and influence each other (Oliveira et al., 2024). Protecting animal health is fundamental to preventing zoonotic diseases, especially in regions of high biodiversity and intense human-animal interaction, such as the Pantanal (Jones et al., 2008), where people, domestic animals and wildlife share the same environment. Detection of zoonotic threats, such as the spread of parasites resulting from environmental degradation and urban expansion, becomes essential (Daszak et al., 2000).

In this context, apex predators are directly linked to the One Health approach. The absence of top predators, such as jaguars, can result in uncontrolled prey population growth. This can trigger ecological imbalances and increase interaction between wildlife and humans, which may favor the spread of zoonotic diseases (Estes et al., 2011). Some parasites found in jaguars or their prey can infect humans, potentially representing a public health risk (Coelho et al., 2011).

Moreover, reduction of the population of large predators may also favor the establishment of invasive species (Estes et al., 2011). One relevant example is the feral pig (*Sus scrofa*), an invasive species in the Pantanal that serves as a reservoir for zoonotic pathogens, including those that cause leptospirosis, toxoplasmosis, brucellosis and foot-and-mouth disease (Pedrosa et al., 2021), and *Spirometra* spp. (Bengtson & Rogers, 2001). Carnivores such as jaguars can act as bioindicators of environmental health, given that the diversity of their diet can reflect the presence of pathogens at different trophic levels (Cleaveland et al., 2006). A wide range of parasites has already been identified in *Panthera onca*, including trematodes (*Alaria* spp., *Paragonimus* spp., *Platynosomum illiciens*, etc.), cestodes (*Diphyllobothrium trinitatis*, *Echinococcus oligarthrus*, etc.), nematodes (*Ancylostoma caninum*, *Toxocara cati*, *Physaloptera anomala*, etc.), and acanthocephalans (*Oncicola oncicola*, *Oligacanthorhynchus pardalis*, etc.), as compiled by Panti-May et al. (2024).

Felids and canids can serve as definitive hosts of cestodes of the genus *Spirometra* (Cestoda: Diphyllobothriidea), in which the parasite completes its maturation and reproduction (Taylor et al., 2016a). The life cycle of these cestodes occurs in semi-aquatic environments and involves intermediate hosts such as copepods, amphibians, reptiles and birds, and other mammals that act as intermediate and paratenic hosts (Bowman, 2010). Sparganosis is a zoonosis caused by ingestion of the larval form (spargana) of *Spirometra* spp., which can manifest in humans in cutaneous, ocular, visceral or cerebral forms (Kuchta et al., 2021). Recent molecular studies have revealed at least seven distinct *Spirometra* lineages worldwide, with evidence of broad host specificity at both definitive and intermediate host levels, although more data are needed to clarify their distribution and diversity. Among them, *S. mansoni* stands out as the only cosmopolitan species, while most other taxa appear to be restricted to specific continents (Kuchta et al., 2024).

The parasite's eggs are eliminated in the feces of felids and ingested by copepods (*Cyclops*), where they develop into the procercoid stage in water. The copepods are then ingested by intermediate hosts, where the procercoids transform into plerocercoids, which are transmitted to predators upon consumption (Bowman, 2010). Human infection occurs mainly through ingestion of water contaminated with infected copepods or of undercooked meat from secondary hosts (Taylor et al., 2016a).

Investigating definitive hosts and identifying risk areas are essential for the prevention and control of zoonotic transmission, especially in regions where interaction between humans and wildlife is intense. Therefore, using the jaguar as an indicator of the parasite’s presence may be useful for understanding the transmission cycle in areas with human presence.

**Figure 1:**
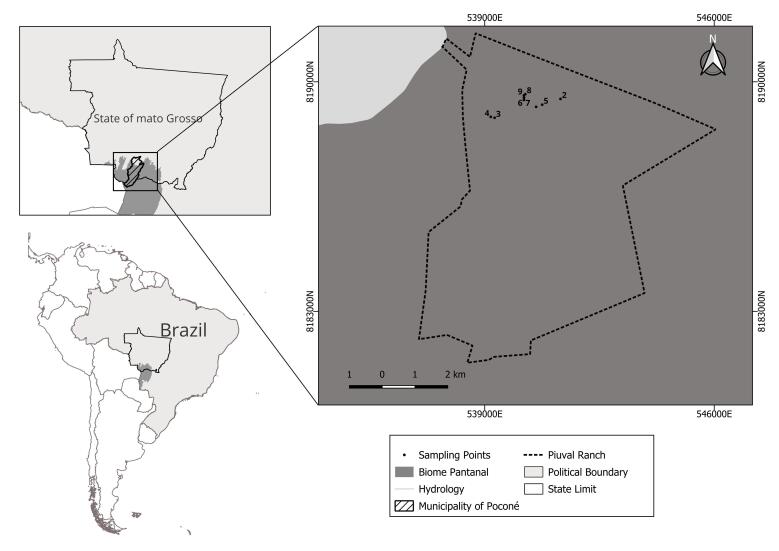
Map showing the sites (1-10) where fecal samples were collected at Piuval Lodge, municipality of Poconé, in the Brazilian Pantanal, state of Mato Grosso, Brazil.

The study area is located at Piuval Lodge, in the municipality of Poconé, in the northern Pantanal of Mato Grosso, in the Central-Western region of Brazil. Piuval Lodge covers an area of 7,200 hectares and is situated approximately 8 km from the town of Poconé (Figure 1). The property is surrounded by several ranches with considerable presence of domestic animals, including cattle, horses, dogs, pigs, poultry and cats. Additionally, the region has a significant population of jaguars, as evidenced by frequent sightings and predation attacks on domestic animals.

**Figure 2:**
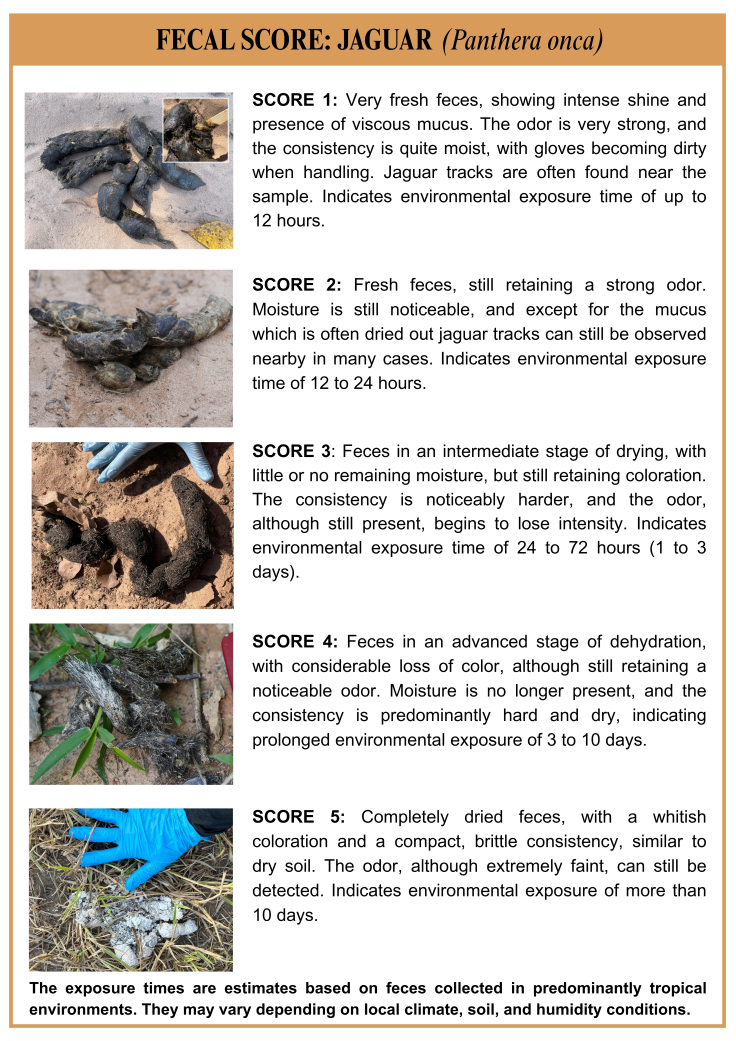
Scorecard illustrating the parameters and morphological characteristics used to classify fecal samples into five categories based on their degree of freshness, in order to estimate their probable origin from jaguars (*Panthera onca*).

Between June and September 2024, 21 fecal samples were collected from the ground, among which 10 were selected based on three criteria: direct observation of the animal during defecation, morphological characteristics and freshness of the samples. Three of these were collected immediately after defecation. The remaining samples were identified through morphological characteristics such as footprints, size and presence of hair and prey bones (Figure 2), following the protocol described by Chame (2003). Only fresh samples were considered for collection, to ensure greater effectiveness in conducting coprological tests (Taylor et al., 2016b).

All samples were collected using disposable gloves, photographed to assess fecal score and georeferenced using the Gaia GPS mobile application (*Gaia GPS Premium*) installed on an iOS device. The date and time of collection, consistency, appearance, odor and location were recorded. From each sample, 5 g aliquots were collected, placed in Falcon tubes containing 10% formalin solution at a 1:10 ratio and stored until processing. The samples were sent to the Veterinary Parasitology Laboratory.

Three coproparasitological techniques were performed: Faust's centrifugal-flotation technique (Faust) (Faust et al., 1938), simple flotation (Willis-Mollay) (Willis, 1921) and spontaneous sedimentation (Hoffman, Pons & Janer, HPJ) (Hoffman et al., 1934). The Faust technique was carried out by diluting 1 ml of fecal material in 10 ml of distilled water, homogenizing the suspension, filtering it and then centrifuging it at 1700 rpm for 1 minute. The supernatant was discarded after washing with distilled water until it became clear. Then, zinc sulfate solution (density of 1.20) was added to the sediment and this mixture was centrifuged again at 1700 rpm for 1 minute. After a 5-minute rest, the material was collected with a platinum loop, Lugol’s iodine was added, and the material was examined by means of light microscopy at 100x, 200x and 400x magnifications to identify parasitic forms.

The Willis-Mollay method was performed by diluting 2 grams of feces in 20 ml of saturated salt solution (NaCl) with a specific density of 1.20. The suspension was homogenized and filtered, and the filtrate was transferred to a test tube until a meniscus formed. The flotation process was then allowed to occur for 10-15 minutes. The coverslip was carefully removed, a drop of Lugol’s iodine was added, and a coverslip was placed in a microscopic slide. The sample was examined under a light microscope at 100x, 200x, and 400x magnification to identify nematode eggs and protozoan cysts and oocysts.

Lastly, the simple sedimentation method of Hoffman was carried out by diluting 2-4 grams of feces in added water, in a glass container. The suspension was homogenized with a glass rod and filtered into a conical sedimentation glass. The volume of the glass was completed with water and the mixture was homogenized again. After a period of 2-3 hours, the sedimented content was collected using a Pasteur pipette. Two drops of the sediment were then transferred to a microscope slide and a coverslip was placed over it. The slide was examined under a light microscope at 100x, 200x and 400x magnifications to identify eggs and other parasitic forms. Images were obtained using a Leica ICC50E camera coupled to a Leica DM 500 microscope.

All 10 samples analyzed showed eggs that were morphologically compatible with *Spirometra* spp.. Samples 1, 2, 3, 4, 6 and 10 were positive through all three methods. In sample 1, in addition to *Spirometra* spp., *Toxocara* spp. eggs were also observed using the Willis-Mollay technique, while sample 3 also showed eggs compatible with *Ancylostoma* spp. using the Faust method. Samples 5, 7 and 8 were analyzed only using the Faust and HPJ methods due to the small amount of material available, and *Spirometra* spp. was detected. Sample 8 was negative through Faust but positive through the sedimentation technique. Sample 9 was positive for *Spirometra* spp. and *Toxocara* spp. through the Faust and Willis-Mollay techniques, but negative through HPJ sedimentation.

Samples 3 and 4 came from a juvenile male jaguar that was observed defecating, and sample 10 came from an adult female also observed at the time of defecation. The remaining samples were identified as jaguar feces based on morphological criteria. All samples that were attributed visually exhibited size, shape, odor and content typical of jaguar feces (Chame, 2003). They were found on trails or hunting areas, often associated with recent tracks or signs of predatory activity, thus reinforcing their attribution to the target species.

The HPJ method demonstrated the highest sensitivity (9 out of 10 samples positive), followed by the Willis technique (limited to 7 samples due to material shortage). On the other hand, the Faust method showed variable results among aliquots.

**Figure 3:**
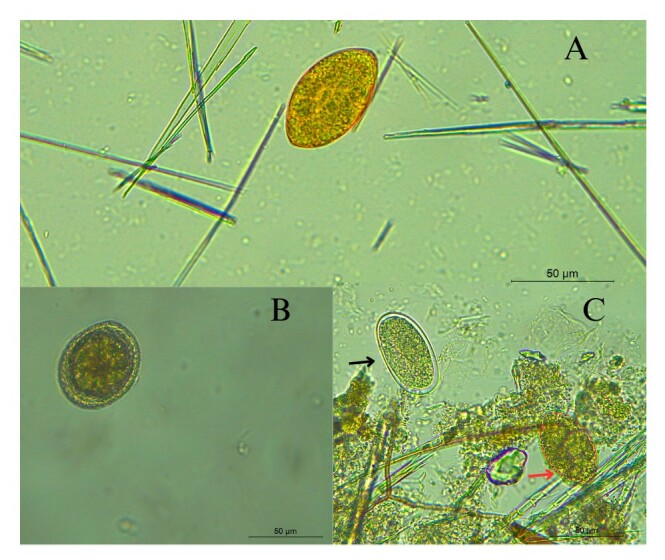
Evolutionary forms of gastrointestinal parasites found through coproparasitological examination. Egg of *Spirometra* spp. (A). Eggs of *Toxocara* spp. (B). Eggs of *Ancylostoma* spp. (black arrow) and *Spirometra* spp. (red arrow) (C), in mixed infection. Staining with a drop of 2% Lugol, scale bar = 50 µm.

Coprological analysis via light optical microscopy revealed eggs compatible with *Spirometra* spp., characterized by an undifferentiated embryo and yolk cells that entirely fill the interior. The yellowish-brown, operculated eggs are asymmetrical along their longitudinal axis and resemble those of trematodes, with the operculum located at one extremity of the shell (Zajac & Conboy, 2012) (Figure 3A). The recovery of *Spirometra* spp. eggs, even in samples with limited material or in different aliquots, suggests that this parasite is widely disseminated. The complementarity between the diagnostic methods enabled broader detection and highlighted the importance of multiple approaches for increasing the sensitivity of parasitological identification.

This is the first report of the presence of *Spirometra* spp. in jaguars in the Brazilian Pantanal. Despite the lack of previous records in the region, this parasite has already been identified in wild felids in other Brazilian biomes, such as the Cerrado (Moraes et al., 2024), and also in several Latin American countries, including the Atlantic Forest of Misiones (Argentina), Uruguay, Peru, Colombia, Mexico, Belize and Bolivia, as well as in captive specimens of jaguars in Brazil and the United States (Arrabal et al., 2020; Panti-May et al., 2024). Human infection is mainly associated with the ingestion of water containing infected copepods or undercooked meat (Taylor et al., 2016a), but additional routes have been described, such as ingestion of raw amphibians and reptiles or the use of animal tissues as poultices in traditional medicine (Kuchta et al., 2021). These practices explain cases reported in Asia (Bowman, 2010; Kuchta et al., 2021), while in the United States feral pigs (*Sus scrofa*) have been recognized as important intermediate hosts, with human cases linked to the consumption of their meat (Bengtson & Rogers, 2001; Taylor et al., 2016a). In South America, however, transmission seems to occur primarily within wildlife, with amphibians and reptiles suspected to act as intermediate or paratenic hosts, although this remains poorly investigated (Arrabal et al., 2020).

The wide hunting ranges and opportunistic feeding habits of jaguars and other large felids increase their exposure to prey species that may harbor plerocercoids, thereby contributing to the maintenance of *Spirometra* in natural environments. As apex predators, they are particularly prone to accumulate and disseminate parasitic agents (Moraes et al., 2024). The presence of zoonotic parasites in wild felids is also a relevant indicator of the interface between predators, their prey, and human populations. Increased interactions among wild species, associated with human activity in natural areas, have been suggested to contribute to the emergence and spread of pathogens with zoonotic potential (Jones et al., 2008). However, the occurrence of *Spirometra* is far more expected in wild carnivores than in humans, with felids being among the main definitive hosts worldwide and in South America, usually without showing clinical signs (Kuchta et al., 2021). In contrast, human infection is rare, but in exceptional cases it can develop into a proliferative disease with severe or even fatal outcomes (Taylor et al., 2016a).

Finally, unresolved aspects of the taxonomy of the genus *Spirometra* must still be considered when interpreting epidemiological data. Likewise, the etiology and variable pathology of sparganosis remain poorly understood, largely because few initiatives have applied molecular and phylogenetic approaches for accurate pathogen identification (Kuchta et al., 2021). In fact, the taxonomy of the group has been regarded as highly problematic, with several species described only from larval stages (*spargana*) that should be treated as *species inquirenda* or even *nomina dubia* unless supported by molecular or life-cycle evidence. Molecular data have already revealed at least seven distinct *Spirometra* lineages, highlighting both the gaps in current classification and the urgent need for integrative approaches combining morphological and molecular evidence (Kuchta et al., 2024).

Importantly, Moraes et al. (2024) also reported *Spirometra* in other wild carnivores such as *Leopardus pardalis* in the Pantanal of Poconé (Mato Grosso), emphasizing that this phenomenon is not restricted to jaguars. Such findings broaden the ecological perspective of parasite circulation in Neotropical felids and suggest that transmission dynamics in these ecosystems may be more complex and interconnected than previously assumed.

Although the main focus of this investigation was the detection of *Spirometra* spp., eggs compatible with parasites of the genera *Toxocara* and *Ancylostoma* were also identified in three of the samples analyzed. Despite limitations on species-level identification, these groups include parasites relevant to public health (Taylor et al., 2016a). These findings reinforce the importance of the One Health approach in preventing disease transmission and conserving wildlife, such that the jaguar can potentially serve as a sentinel species for zoonotic monitoring (Thornton et al., 2016).

Given this scenario, the jaguar stands out as a potential bioindicator of environmental health and a sentinel species for zoonotic monitoring in the Pantanal. Identification of parasites through noninvasive techniques, such as coprological analysis, reveals not only the health status of wild fauna but also emphasizes the importance of the One Health approach in disease prevention and biodiversity conservation.

With the close coexistence between wildlife and livestock in the region, understanding the parasitic load of jaguars is essential for promoting coexistence strategies and protecting public health. Future studies will be fundamental for deepening knowledge of the transmission cycles of *Spirometra* spp. and other parasites and for proposing effective mitigation measures.
